# Interictal Neurocognitive Processing of Visual Stimuli in Migraine: Evidence from Event-Related Potentials

**DOI:** 10.1371/journal.pone.0080920

**Published:** 2013-11-14

**Authors:** Marla J. S. Mickleborough, Christine M. Chapman, Andreea Simina Toma, Jeremy H. M. Chan, Grace Truong, Todd C. Handy

**Affiliations:** 1 Psychology Department, University of Saskatchewan, Saskatoon, Saskatchewan, Canada; 2 Psychology Department, University of British Columbia, Vancouver, British Columbia, Canada; 3 Dartmouth College, Hanover, New Hampshire, United States of America; University Medical Center Goettingen, Germany

## Abstract

Research has established decreased *sensory* habituation as a defining feature in migraine, while decreased *cognitive* habituation has only been found with regard to cognitive assessment of the relative probability of the occurrence of a stimulus event. Our study extended the investigation of interictal habituation in migraine to include cognitive processing when viewing of a series of visually-complex images, similar to those we encounter on the internet everyday. We examined interictal neurocognitive function in migraine from a habituation perspective, using a novel paradigm designed to assess how the response to a series of images changes over time. Two groups of participants--migraineurs (N = 25) and non-migraine controls (N = 25)--were asked to view a set of 232 unfamiliar logos in the context of a target identification task as their brain electrical responses were recorded via event-related potentials (ERPs). The set of logos was viewed serially in each of 10 separate trial blocks, with data analysis focusing on how the ERP responses to the logos in frontal electrodes from 200-600 ms changed across time within each group. For the controls, we found that the amplitude of the late positive potential (LPP) ERP component elicited by the logos had no significant change across trial blocks. In contrast, in migraineurs we found that the LPP significantly increased in amplitude across trial blocks, an effect consistent with a lack of habituation to visual stimuli seen in previous research. Our findings provide empirical support abnormal cognitive processing of complex visual images across time in migraineurs that goes beyond the sensory-level habituation found in previous research.

## Introduction

Migraine is a primary headache disorder which is strongly associated with dysfunction of neuronal cortical excitability in between headache attacks [1]. There is debate as to whether the migraine sensory cortices should be described as hyperexcitable [1] or as hyperresponsive – to reflect that the cortex seems to react excessively to repetitive, not single, stimuli [2]. Either way, strong evidence for this dysfunction includes heightened visual sensitivity between migraine attacks [1,3-7], and interictal deficits in sensory habituation to repetitive stimuli, as revealed by EEG-based measures of evoked cortical responses [8]. Specifically, whereas non-migraineurs show reductions in the amplitude of visual-evoked components to repeated stimuli over time, migraineurs show no evidence of this time-based sensory attenuation [8-12]. Thus, consistent research support the hypothesis that interictal visual sensitivity and decreased sensory habituation are hallmarks of migraine. 

While impaired sensory habituation in visual cortex in migraine is now well-documented, there has been little research assessing the impact beyond the early sensory level of response, towards cognitive issues surrounding the interictal hyperexcitable visual cortex and impaired habituation in the everyday life of a migraineur. Specifically, whether the cortical hyperexcitability and habituation deficits may be influencing the neurocognitive processing of stimulus events and thus affecting daily cognition in migraineurs is still unclear. Indeed, while migraineurs frequently have interictal cognitive complaints such as heightened sensitivity to extraneous sensory inputs and general difficulties with focusing attention [13-17], empirical attempts to assess cognition in migraineurs have been inconsistent, leading many researchers to conclude there are no interictal cognitive abnormalities in migraine. For example, a recent review of the literature reveals only mixed evidence for interictal neuropsychological impairments in migraine populations and states that migraine has only “a trivial effect in the cognitive domains of processing speed, attention, verbal learning and recall, working memory, sustained attention, and inhibition” [18]. The authors then suggest that the inconsistent findings may be due to cognitive impairments only being in a subset of migraineurs [18]. While this is a possibility, it may be that the inconsistent findings are due to a disconnect between the known abnormaliies in interictal sensory processing (ie, visual sensivities and lack of sensory habituation) and what neuropsychological assessments have typically targeted for study--broad cognitive assessments of verbal and memory abilities, motor and visuospatial skills, reasoning, and executive control functions [18]. In other words, it makes sense to look for cognitive abnormalities that specifically target cognitive processing that would likely to be impacted by hyperexcitable visual cortices.

Consistent with this conclusion, the most reliable evidence to date of interictal cognitive deficits in migraine has come from two domains of research, both investigating aspects of cognition likely sensitive to hyperexcitable visual cortical responses. First, attenuation of sensory responses in cortex is essential to normal selective attentional function [19], yet migraineurs manifest heightened attentional orienting to peripheral sensory inputs [20,21], an effect that may be tied to a reduced ability to attenuate responses to unattended events [22]. Second, event-related potential (ERP) measures have shown habituation deficits in migraineurs in two components which index the implicit analysis of whether or not a particular stimulus is expected to occur - the contingent negative variation (CNV) [23,24] and the P3 ERP components [12,25,26]. Specifically, whereas non-migraineurs will show reductions in the amplitude of these components in response to infrequent red flashes of light amongst frequent white flashes of light over the course of a testing session, migraineurs show evidence of stable or increasing amplitudes over time to these same “oddball” stimuli [27-29]. Collectively, these findings have established decreased *sensory* habituation as a defining feature in migraine, while decreased *cognitive* habituation has only been found with regard to cognitive assessment of the relative probability of an infrequent oddball event (a red flash of light) amongst more frequent "standard" events (white flashes of light) [27–29]. 

The goal of our study was to investigate whether interictal habituation deficits in migraine extend beyond the quantitative analysis of event probabilities to cognitive processing when viewing of a series of more visually-complex images, similar to those we encounter on the internet everyday. Even with only a simple cognitive task such as discriminating a stimulus as a target or non-target, people have been shown to evaluate visual images at an implicit cognitive level [30]. As such, our stimuli moved beyond the sensory-level response to checkerboard reversals [8-12] and cognitive-level response to “oddball” paradigms [27-29] previously used to study habituation in migraineurs, to look at more ecologically valid images – in this case, serial presentation of commercial branding logos. Using such stimuli, Handy et al. [30] compared the amplitude of the late positive potential (LPP) ERP component elicited by logos across the eight trial blocks used in their study, and found that the waveform amplitude rapidly stabilized with repeated stimulus exposure--from the second trial block on, there was no significant change in amplitude across blocks in the LPP. This suggested that after initial exposure to the stimulus set, the depth of post-sensory neurocognitive processing of the logos did not change over time. As such, our analysis focused on two issues: (1) Could we replicate this neurocognitive habituation effect in a set of non-migraine controls, and (2) Would migraineurs show an absence of this habituation effect, and instead manifest an increase in LPP amplitude across trial blocks, similar to results found in other habituation studies of migraine? If so, this research will be a natural extension to the vast literature showing that migraineurs have decreased habituation of visual-evoked potentials and event-related probabilities to encompass cognitive processing of complex visual images. 

## Materials and Methods

To investigate whether interictal habituation deficits in migraine extend to cognitive processing of complex visual images, we had participants view common everyday branding logos. These stimuli that have been shown in a recent study to be automatically and implicitly processed at a cognitive level – specifically, without conscious intention, individuals categorize the images as liked or disliked, and these preferences are represented in brain response in as little as 200 msec after image viewing [30]. We asked both migraineurs and non-migraine controls to view a serial stream of 232 different and unfamiliar logos in the context of a target identification task while their ERP responses were recorded. In each trial block, each of these 232 logos was presented exactly once, while a target logo was presented 20 times, and no explicit instructions were given to consciously evaluate or assess the aesthetics of the logos themselves. A total of 10 trial blocks were performed by each participant, with block serving as our temporal unit of measure for examining between-group changes in cognitive habituation when viewing complex visual images.

### Participants

50 paid volunteers participated; 25 were in the non-migraine control group (16 women and 9 men; age 25.8, SD 11.7) and 25 were in the migraine group (16 women and 9 men; age 26.1, SD 8.7). The migraine group included individuals with aura (n=11) and without aura (n=14), and the migraineurs averaged 19.4 (SD 25.1) headaches a year, with the average headache lasting 8.0 hrs (SD 7.2). On average, our migraine participants had been having migraines for 10.1 (SD 8.1) years. None of our participants were taking migraine prophylactics. All participants were at least 18 years old and gave written informed consent to participate in the study and all testing procedures were approved by the University of British Columbia Clinical Review Ethics Board (H07-00458).

### Headache Classification

All migraine participants were required to meet the migraine criteria specified by the International Headache Society [31] and as determined by an interview. Because migraine habituation effects are thought to normalize prior to and during an attack [32], all migraineurs had not had a migraine within 48 hours prior and 48 hours after the testing period (confirmed via email follow-up). In addition to our headache classification criterion, migraineurs were excluded if they were taking any form of migraine prophylactics. Migraineurs were recruited via posters in the university community.

### Stimuli

This stimulus set was adapted from Handy et al. [30]. A total of 232 non-target logos were used as the primary stimulus set with one target logo. All logos can be viewed at (http://attention.psych.ubc.ca/Site/Downloads.html). The logos were drawn from sources publicly available on the Internet. Criteria for inclusion in this set included that the logo contained no verbal/lexical information (i.e., no words or letters) and that it was not a widely known or familiar image (e.g., such as the Nike "swoosh"). Post-experiment debriefing was used to determine whether any of the logos were previously familiar or known. If any logos were recognized, the data from epochs containing responses from the familiar logos were exluded in that participant’s data set. 

### Procedures

Each trial block began with the presentation of the target logo for 2 s as a reminder of which logo required a manual response to be made; the same logo was used as the target across all trial blocks and participants. Within each trial block, this target was presented 20 times and each of the 232 non-target logos were presented once, with the order of presentation randomly varied between 10 trial blocks. The duration of each stimulus was 200 ms (with a standard frame rate of 60Hz, for 12 frames of stimulus presentation), and the inter-stimulus interval was randomly varied between 1300-1500 ms. Stimuli were presented on a VGA monitor controlled by a Pentium PC using the VAPP stimulus presentation system (http://nilab.psychiatry.ubc.ca/vapp/), and manual responses to the target were made by pressing a button on a hand-held joystick, with the thumb of response (left vs. right) counterbalanced between participants. Instructions to the participants asked them to simply observe the logos on the screen and make a manual response as quickly as possible whenever the target logo was presented. No instructions were given to think about or explicitly evaluate the non-target logos.

### Electrophysiological Recording

Scalp potentials were recorded from 64 Ag/AgCl active electrodes via a Biosemi Active-Two ERP amplifier system. To ensure proper eye fixation and allow for the removal of events associated with eye movement artifacts, vertical and horizontal electro-oculograms (EOGs) were also recorded – the vertical EOG from an electrode inferior to the right eye, and the horizontal EOG from an electrode on the right outer canthus. Two additional electrodes were used to record from the left and right mastoids. Data were recorded relative to Active-Two's CMS/DRL feedback loop (Common Mode Sense [CMS] and Driven Right Leg [DRL] - which replace the ground electrode in conventional systems), using a second order low-pass filter of 0.05 Hz with a gain of 0.5 and with a digitized on-line sampling rate of 256 samples-per-second. Offline, all scalp electrodes were referenced to the average of the left and right mastoid signals. Automated artifact rejection was then used to eliminate trials with detectable eye movements, blinks, muscle potentials or amplifier blocking. An average of 9.8 events were dropped for eye movements in each block, with no significant difference between groups (10.4 (SD 17.31) for migraineurs and 9.16 (SD 12.44) for controls, t(48)=0.29, *p* = 0.77). 

For each participant, the waveforms time-locked to the remaining events of interest were epoched into 800 ms segments, beginning 200 ms before stimulus onset until 600 ms post-stimulus. These single-subject waveforms were then used to generate the group-averaged waveforms for display and analysis. A -200 to 0 ms pre-stimulus baseline was used for all ERP waveform measurements and displays. Planned ERP data analysis focused a priori on the mean amplitude across 200-400 ms and 400-600 ms post-stimulus windows elicited at frontal/central electrode sites in order to replicate the “repeated stimulus exposure” analysis by Handy et al. [30]. The timing of these late ERP responses are consistent with implicit cognitive processing. Specifically, in these late time windows, cognitive processing such as attention, memory, categorization, goals, etc., have had time to influence the sensory processing that occurs and are separable in the neurocogntive electrophysiological literature from the earlier basic sensory responses to stimuli [33,34]. These windows are also consistent with the time-ranges found to be sensitive to visual cognitive habituation in previous migraine studies [27-29].

## Results

### Behavior

 Both groups were highly accurate at detecting the 20 target logos for each block, with no significant difference between mean target detection accuracy of 98.42% (SD = 2.7) for controls and 98.83% (SD = 1.6) for migraineurs (F(1,48) = 0.427, *p* = 0.517). Speed of detection also did not differ between groups (F(1,48) = 0.243, *p* = 0.624), with mean reaction times of 451.4 (SD = 53.9) for controls and 458.8 (SD=52.3) for migraineurs.

### ERP responses to logos across trial blocks

**Figure 1:**
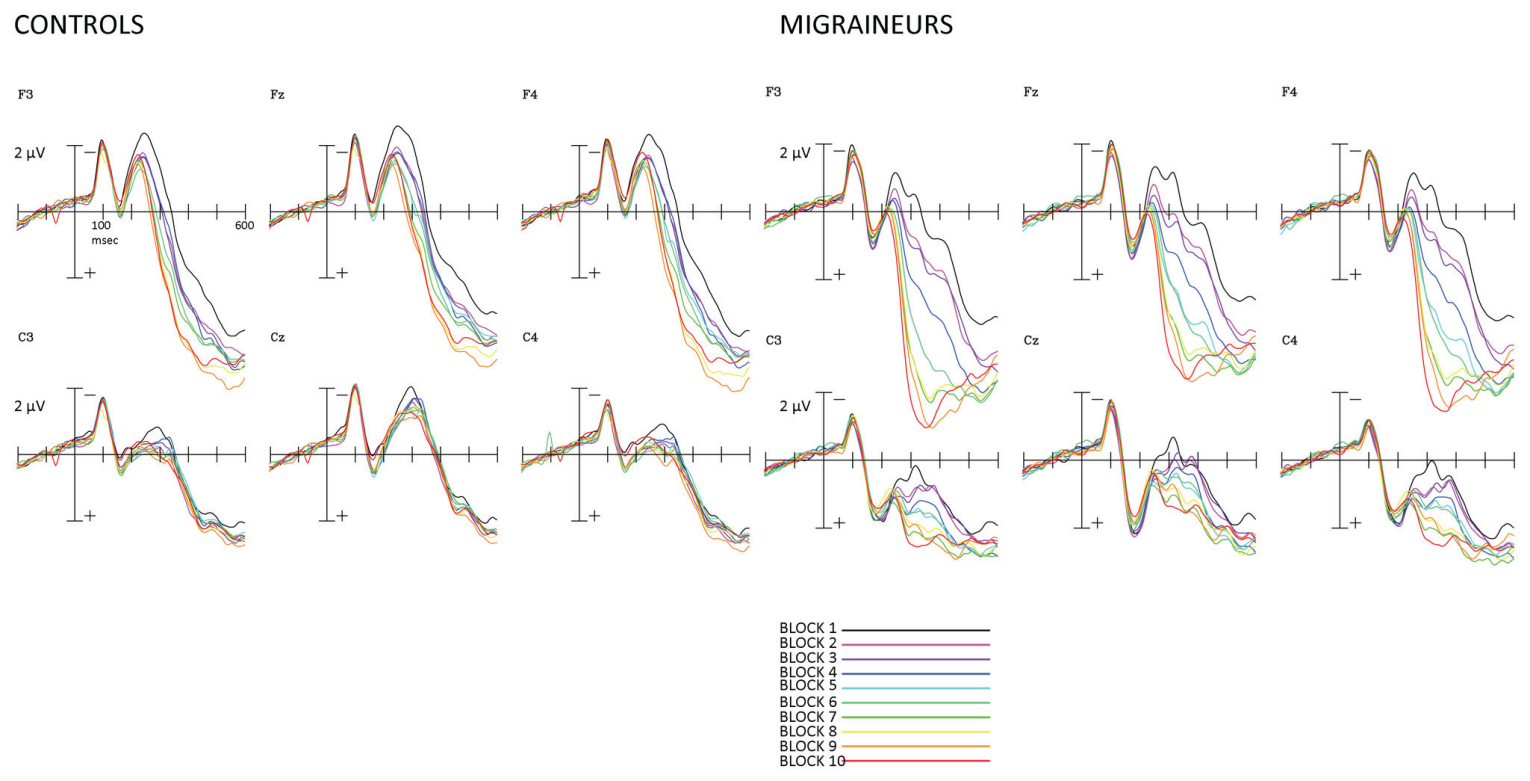
Grand-averaged ERP waveforms as a function of group, block, and scalp location. Control Group (N=25). Migraine Group (N=25). Shown are frontal-central electrodes F3, FZ, F4, C3, CZ, C4 with first block (black line) through to 10^th^ block (red line).

**Figure 2:**
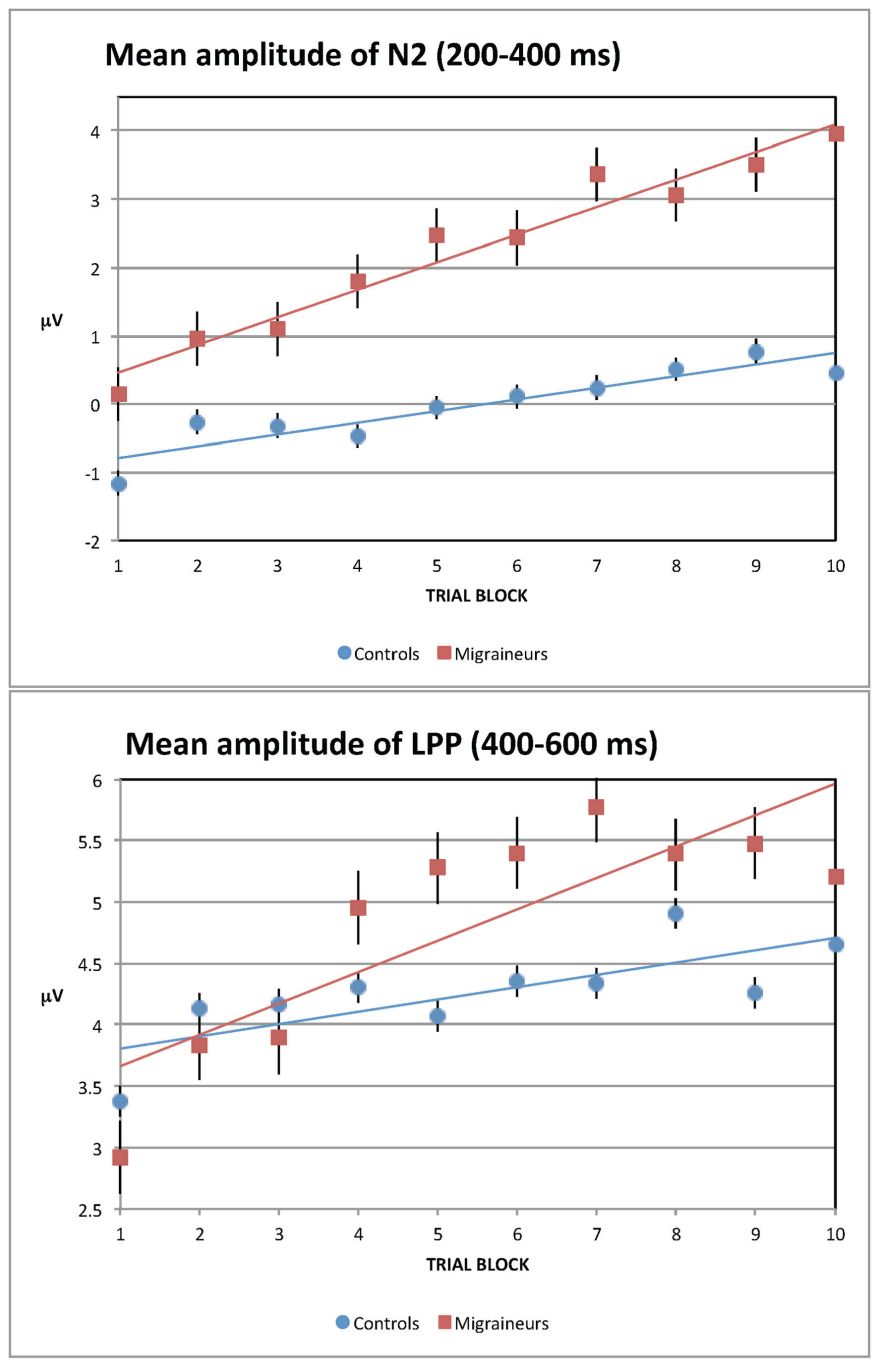
Grand-averaged mean amplitudes as a function of group, block, and time window (a. 200-400 ms; b. 400-600 ms), averaged across frontal-central electrodes F3, FZ, F4, C3, CZ, and C4. Control Group (N=25). Migraine Group (N=25).

 As can be seen in Figure 1, the amplitude of the late positive-going deflection in the waveform appeared to increase across trial blocks starting around 250 ms in the migraineurs but not controls. This data pattern was confirmed via an omnibus repeated measures ANOVA that included a between-subjects factor of group (control vs. migraine), and within-subjects factors of trial block (blocks 1-10), time window (200-400 ms vs. 400-600 ms), and location, which was split across two factors, frontal vs. central recording site, and hemisphere of recording: left (F3/C3) vs. right (F4/C4) vs. midline (FZ/CZ). Because the electrode factors were of non-interest, we do not report any main effects or interactions involving either scalp location or hemisphere of recording. Mean amplitudes for each time window are shown in Figure 2 as a function of group and block, and collapsed for electrode. We found a significant group x trial block interaction (*F*(2,48) = 2.93; *p* < 0.01), and a significant group x trial block x time window interaction (*F*(2,48) = 1.44; *p* < .05). There were also main effects of group (*F*(2,48) = 13.24; *p* < 0.001), and trial block (*F*(2,48) = 5.90; *p* < 0.001), but there was no main effect of time window (*F*(2,48) = 2.78; *p* = 0.07).

The pair of significant interactions involving group were then followed-up via separate planned repeated-measures ANOVAs within each group and time window. This confirmed that in migraineurs there was effect of block in both the 200-400 ms window (*F*(1,24) = 3.94; *p* < 0.001) and the 400-600 ms time window (*F*(1,24) = 4.03, p < 0.001). In contrast, there were no significant effects of trial block in the controls in either time window (200-400 ms, *F*(1,24) = 1.66; *p* = 0.10; 400-600 ms, *F*(1,24) = 0.89; *p* = 0.54). 

 Given the initial findings of block-related effects on the amplitude in migraineurs but not controls, we wanted to more precisely characterize the nature of this effect. To further explore this interaction, for each group we used a regression analyses to test whether block was significantly predictive of the mean amplitude for each of the time windows, collapsed across electrodes. As highlighted by the regression lines in Figure 2, we found that for migraineurs block was predictive in both the 200-400 ms time window (*b* = 0.40, *t*(8) = 12.93, *p* < 0.001), indicating each increase in block predicted a .40 μV increase in mean amplitude, and in the 400-600 ms time window (*b* = 0.26, *t*(8) = 17.71, *p* = 0.003), indicating each increase in block predicted a 0.26 μV increase in mean amplitude. In comparison, for controls block was also significantly predictive of the mean amplitude of both the 200-400 ms time window (*b* = 0.17, *t*(8) = 6.58, *p* < 0.001) and the 400-600 ms time window (*b* = 0.10, *t*(8) = 10.93, *p* = 0.011), but these effects were predicted only a 0.17 and 0.10 μV increase in mean amplitude, respectively. Finally, this apparent between-group difference in predicted amplitude change was confirmed statistically, where block was significantly more predictive of an increase in mean amplitude in migraineurs relative to controls for both the 200-400 ms time window (*t*(16) = 5.71, *p* < 0.001), as well as for the 400-600 ms time window (*t*(16) = 2.29, *p* = 0.036). 

 In order to examine possible covariate influences of age, gender, or clinical parameters, we ran a multivariate ANCOVA. As there were no significant effects of age, gender, frequency of attacks, duration of attacks, years with headache, or severity of headache on the ERP measures (all *F*s < 2.063, all *p*s > 0.166), we did not include these covariates in the main results.

## General Discussion

Substantial evidence indicates that migraineurs have a deficit of sensory-level habituation to repetive visual-evoked stimuli [8-12]. Evers and colleagues have shown this effect extends beyond the sensory response, revealing that migraineurs manifest an increase in ERP activation to stimulus expectancies [27-29]. In this study, we extend this theory by showing that migraineurs lack habituation when viewing a series of commercial branding logos such as those found on websites on the internet. Specifically, whereas the amplitude of the late positive potential (LPP) ERP component elicited by logos systematically increased in migraineurs across trial blocks, there was no significant effect of block in controls. Consequently, our results are further support for the hypothesis that impaired interictal habituation is a mechanisms integral to migraine [2], and reveal that interictal habituation deficits in migraineurs are not limited to sensory events [5-9] and stimulus expectancies [27-29], but extend to cognitive processing of complex visual stimuli. 

Our data show clear evidence of group differences across time while viewing complex visual stimuli. What cognitive processes are associated with this group difference in the LPP? While a common finding with the LPP is that emotional (positively and negatively valenced) images show a greater LPP than neutral images, the current explanation of this effect is that highly emotional images lead to a greater motivational attention, and the LPP is increased for motivationally relevant stimuli [35-37]. Indeed, the LPP is also known to be sensitive to top-down manipulations of attention [38]. For example, during viewing of neutral pictures, the LPP of normal control subjects has been shown to attenuate with stimulus repetition, revealing that the LPP reflects attention to the pictures, and attention declines with stimulus repetition [39]. Using the LPP as a measure of motivational attention [38-40], then, given our neutral stimuli, it follows that the increase in LPP across time in the migraineurs in our study may be related to a change in attentional processing across time. Indeed, the idea that migraineurs have abnormal attentional processing fits with previous research in migraine. In particular, findings stemming from a variety of experimental paradigms suggest that, interictally, migraineurs show heightened attention to irrelevant visual stimuli [1,21,22,41-43]. Two key questions follow from our findings and interpretations. First, how might this finding impact migraine research, and second what might this mean for other neuropsychological research?

First, if the LPP is reflecting motivational attention and is increasing across time in migraineurs, what does this mean for migraine research? While previous reports revealed sensory-level habituation effects [8-12], our results suggest the sensitivity to repeated visual stimuli extend to cognitive-level processing of complex images, and that these findings are linked to abnormal attentional processing. This type of subtle cognitive effect is consistent with migraineurs reporting cognitive issues in-between headache events [44]. While one recent review of the literature concluded that clinical efforts to identify cognitive impairments have provided mixed results at best [18], another recent review suggests that patients with migraine in clinics show mild cognitive changes, but that greater methodological refinement is needed to establish whether this cognitive dysfunction appears in community migraineurs [45]. Our findings support this conclusion. Indeed, while traditional neuropsychological tests used in the clinical setting such as trail-making, digit span, verbal fluency and the like are typically designed to assess the functioning of specific neurocognitive systems of interest, they may be insensitive to the intricate ways in which stimuli are attended, processed and evaluated as system-integrating perceptual/cognitive events. Our data, and those of Evers and colleagues [27-29] have shown robust evidence of interictal neurocognitive pathologies in migraine populations using sensitive perceptual-cognitive paradigms, suggesting that there is a benefit to focusing on neurocognitive assessments in migraine that specifically target how stimuli are attended and implicitly evaluated. Our data support the idea that cognitive effects in migraine might be influenced by sublte abnormaliites in attentional processing, and indicates that research needs to continue to focus on understanding the impact of the migraine on day-to-day life in individuals between headache events. 

Second, beyond migraineurs, this research also makes a relevant point for neuropsychological research in general. A key question in our study was whether we could replicate the effect found by Handy et al. [30], such that the LPP waveform elicited by logos stabilized after the second trial block and remained unchanged to the end of eight trials. Our study also found that indeed controls very quickly habituate to the images with no statistically significant effect of block. Importantly, we do not have the migraine status for the Handy et al. control group, but can assume that it contained migraineurs, given that migraineurs make up 7.8% of men and 24.9% of women in Canada [46]. Given our data revealing a change in migraineurs across time, the inclusion of migraineurs in “normal” groups may be contributing some variability to data in other studies. Specifically, researchers who are looking at perceptual and cognitive processing across time could consider including migraine status as a factor in their analyses. We are not suggesting that migraineurs need to be excluded from basic research, but that having a very brief questionnaire screening for migraine may allow this characteristic to act as a covariate and to help control some of the variability found in neuropsychological research. 

### Limitations and Future Directions

This data is compelling and suggests that, across time, migraineurs are responding to everyday visual material in ways that are different than controls. That said, it is important to note that while a *susceptibility* to abnormal attentional responses likely continues across the whole interictal period, the actual change we are seeing occurs across ten trial blocks of repeated exposure to serial presentation of logo images. Future research could test the duration for which this increase in brain response across time continues – in other words, if we were to test for longer periods of time, would the waveforms eventually start to stabilize and would we see the brain response attenuate/habituate in migraine? Perhaps the more interesting question is to continue this extension to even more ecologically valid paradigms, perhaps showing repetition of short videos (such as TV commercials) and seeing whether migraineurs and controls differ in their response across 10 blocks of video presentation. Finally, as our participant group was community-based rather than clinic-based, we did not have information about comorbidities which could be valuable as covariates in analyses. These questions will help us to better understand how the migraine brain is responding to the world on a day to day basis.

## Conclusions

Here we showed that migraineurs lack the normal response of cognitive habituation, as found in controls, when viewing a series of commercial branding logos such as those found on websites on the internet. Previously, lack of sensory-level visual habituation in migraineurs has been described in response to checkerboard reversals [8-12], and a lack of cognitive-level visual habituation in response to “oddball” red flashes amongst frequent white flashes of light [27-29]. Our data extends this lack of habituation to include cognitive processing of more complex visual images, and we are able to clearly show this effect occuring across ten trial blocks.
